# Absent right common carotid artery associated with left accessory middle cerebral artery diagnosed by computed tomography angiography

**DOI:** 10.1007/s00276-025-03658-0

**Published:** 2025-05-24

**Authors:** Atsushi Hashio, Akira Uchino, Kaima Suzuki, Yasutaka Baba, Hiroki Kurita

**Affiliations:** 1 Department of Cerebrovascular Surgery and Stroke Center, International Medical Center, Saitama Medical University, 1397-1 Yamane, Hidaka, Saitama 350-1298 Japan; 2 Department of Radiology, Saitama Sekishinkai Hospital, 2-37-20 Irumagawa, Sayama, Saitama 350-1305 Japan; 3 Department of Diagnostic Radiology, International Medical Center, Saitama Medical University, 1397-1 Yamane, Hidaka, Saitama 350-1298 Japan

**Keywords:** Anatomic variation, Common carotid artery, Computed tomography angiography, Embryology, Internal carotid artery, Middle cerebral artery

## Abstract

**Purpose:**

To describe a case involving congenital absence of the right common carotid artery (CCA) associated with a left accessory middle cerebral artery (MCA) that was diagnosed by computed tomography angiography (CTA).

**Methods:**

A 63-year-old woman with a narrow right internal carotid artery (ICA) incidentally detected on magnetic resonance imaging underwent CTA for further vascular evaluation.

**Results:**

CTA revealed absence of the right CCA, with the right external carotid artery (ECA) branching from the brachiocephalic trunk and the right ICA branching from the right subclavian artery. The ICA was hypoplastic. A left accessory MCA was also observed. Although blood flow in the right ICA was decreased, the patient was asymptomatic; thus, conservative treatment was administered, and her clinical course remained uneventful.

**Conclusion:**

Absence of the CCA is associated with the development of the aorta and various vascular variations; however, to our knowledge, this is the first report of an association with a contralateral accessory MCA, the existence of which may be considered incidental. Preoperative knowledge of this rare variation is important when considering endovascular treatment of cerebral aneurysms and other arterial lesions.

## Introduction

Congenital absence of the common carotid artery (CCA), characterized by separate origins of the internal carotid artery (ICA) and external carotid artery (ECA), is an extremely rare anatomical variation, with fewer than 100 cases reported till date [17]. When this anomaly occurs on the right side, the right CCA is absent; the right ECA branches from the brachiocephalic trunk (BCT) and the right ICA branches from the right subclavian artery (SA).

In early pregnancy, the primitive arterial network regresses and fuses to form the adult arterial system. When this regression and fusion fail, various arterial anomalies occur. Anomalies of the middle cerebral artery (MCA) include an accessory MCA and a duplicated MCA. A duplicated MCA originates from the terminal portion of the ICA and supplies blood to its temporal branch. On the other hand, an accessory MCA originates from the anterior cerebral artery and typically supplies the anterior frontal lobe. The reported prevalence of accessory MCA is between 0.03% and 2.7% [15].

Absence of the CCA is associated with aortic developmental abnormalities, and its association with other vascular variations has been reported [3, 6, 11, 13, 16]. Here we report an extremely rare case involving congenital absence of the right CCA associated with a left accessory MCA that was diagnosed by computed tomography angiography (CTA).

## Case report

**Fig. 1:**
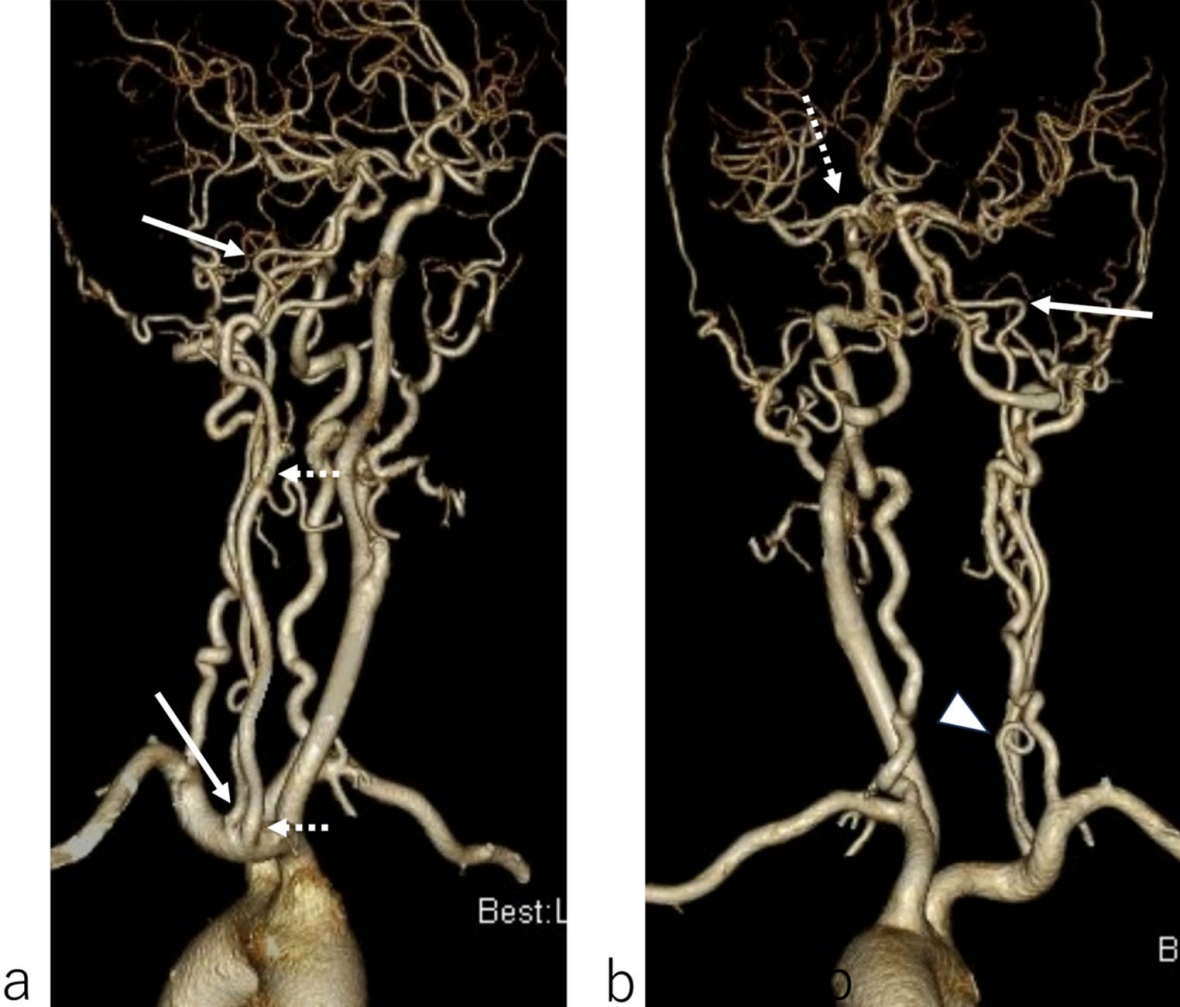
**a**: Right anterior oblique projection of computed tomography angiography (CTA) shows an absent right common carotid artery (CCA). The right external carotid artery (ECA) branching from the brachiocephalic trunk (BCT) (*dotted arrows*), and the right internal carotid artery (ICA) branching from the right subclavian artery (SA) (*arrows*). **b**: Slightly left posterior oblique projection of CTA shows that the right ICA is hypoplastic (*arrow*) and forms a coil (*arrowhead*). A left accessory middle cerebral artery (MCA) (*dotted arrow*) is also seen

**Fig. 2:**
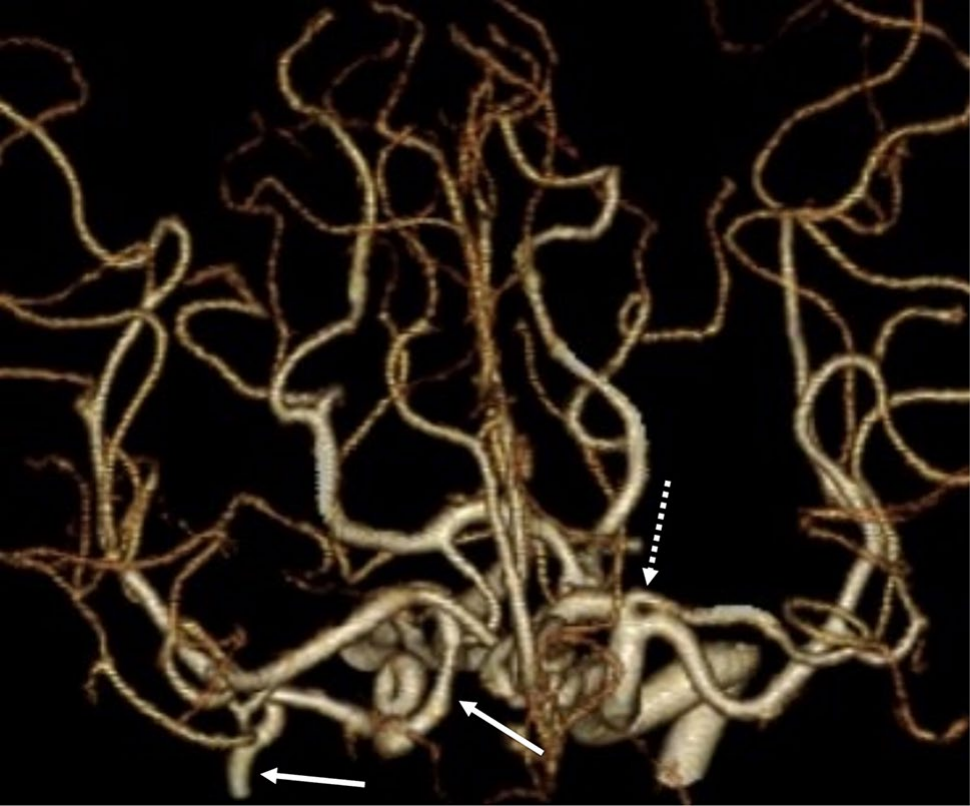
Anterosuperior to posteroinferior projection of CTA of intracranial region shows a left accessory MCA (*dotted arrow*) and a hypoplastic right ICA (*arrows*). Hyperplastic right posterior communicating artery and the right MCA is normal in size

A 63-year-old woman with a narrow right ICA incidentally detected on magnetic resonance imaging underwent CTA for vascular evaluation. She had no medical or family history. CTA showed an absent right CCA, with the right ECA branching from the BCT and the right ICA branching from the right subclavian artery (SA) (Fig. 1a). The ICA was hypoplastic and formed a coil in its proximal segment (Fig. 1b). Intracranial vascular evaluation revealed a left accessory MCA (Fig. 2). Collateral blood flow was observed in the right posterior communicating artery, maintaining adequate perfusion of the right MCA. Although the right ICA was poorly developed, the patient was asymptomatic. Therefore, conservative treatment was administered, and her clinical course was uneventful.

## Discussion

**Fig. 3:**
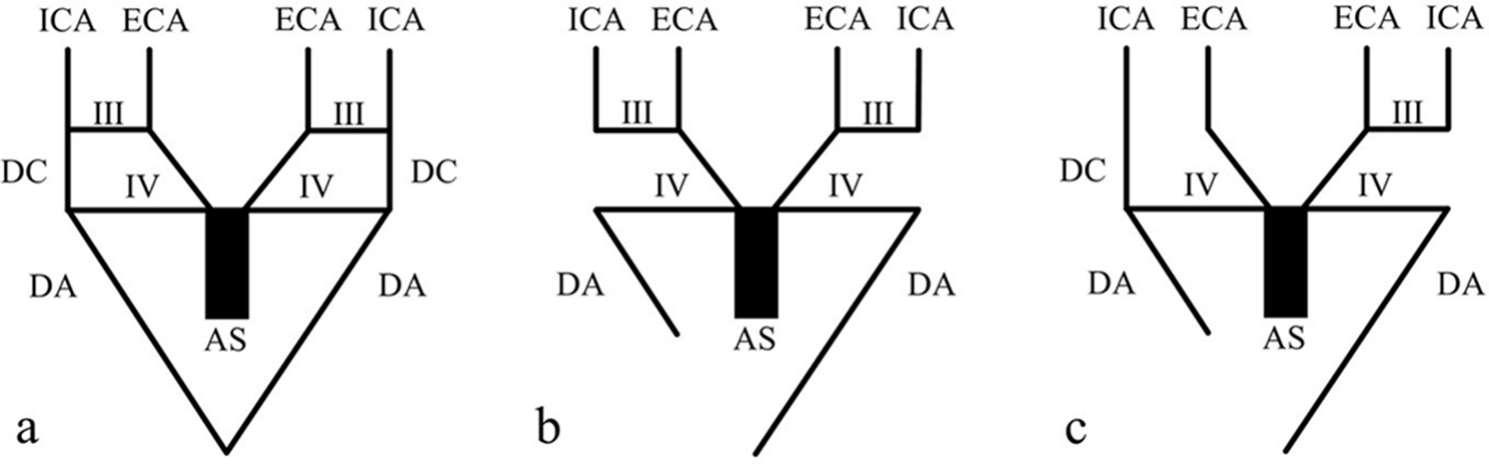
Schematic illustration of absent common carotid artery development. **a**: Six pairs of right and left aortic arches are formed at around 4–5 weeks of the embryonic stage, with the first, second, and fifth aortic arches obliterated early. The sixth arterial arch is omitted. The third and fourth aortic arches are involved in CCA formation. **b**: Normally, the ductus caroticus (DC) obliterates the continuity of the third and fourth aortic arches, and only the third aortic arch is responsible for cephalic nutrition. The fourth aortic arch becomes part of the aorta on the left side and nourishes the trunk. The right side becomes a BCT. The distal portion of the dorsal aorta on the right side is obliterated and the proximal portion become the right SA. **c**: If the right third aortic arch regresses and the DC remains, the ECA branches from the BCT and the ICA branches from the SA, resulting in an absent CCA AS: aortic sac, DA: dorsal aorta, DC: ductus caroticus, ECA: external carotid artery, ICA: internal carotid artery, III: third aortic arch, IV: fourth aortic arch (Modified from [17]).

Congenital absence of the CCA, characterized by separate origins of the ICA and ECA, is an extremely rare anatomic variation, with fewer than 100 reported cases involving the absence of one or both CCAs [17]. In 2014, Berczi et al. [1] reviewed 33 cases involving absence of CCA and reported no significant differences in laterality or sex distribution. Patients remain asymptomatic unless there are other pathologies, and most previous reports have described an incidental presentation [1]. The anomalies in the present case were also discovered incidentally, and the patient was clinically asymptomatic. Absence of the CCA is associated with anomalies in aortic development. During early embryogenesis, six pairs of aortic arches form, with the first, second, and fifth pairs typically regressing. The third, fourth, and sixth arches remodel to form the complete aorta and its branches and the pulmonary arteries (Fig. 3a). The third arches are precursors of the carotid system, while the fourth arches develop asymmetrically. The left fourth arch remains continuous with the aortic arch sac and left dorsal aorta to form the left aortic arch. The right fourth arch, together with part of the right dorsal aorta, forms the BCT and proximal right SA. The dorsal aorta between the third and fourth arches, called the ductus caroticus (DC), regresses by the sixth week [7]. The ECA forms by migration of the ventral pharyngeal artery from the aortic sac to the third arch (Fig. 3b) [10]. Separate origins of the ECA and ICA occur if the DC does not involute and the third arch regresses (Fig. 3c). It has also been reported that the ICA is thinner than normal [4]. In the present case, ICA hypoplasia with a coil-shaped course was observed. A coiling ICA is also an anomaly that occurs during fetal development. During fetal development, as the heart and great vessels migrate to the thoracic cavity, the ICA may continue to extend at an angle if it fails to straighten, resulting in a tortuous, coil-shaped, or kinked configuration [5, 14]. A tortuous ICA refers to an elongated ICA that exhibits exaggerated curves in a C-shaped or S-shaped configuration. A coiling ICA refers to an elongated ICA that forms a circular structure. Kinking is a deformation in a coiling ICA, referring to a state where the cervical ICA extends at an angle of < 90° [14]. The reported prevalence rates for tortuous, coiled, and kinked ICAs are 35%, 6%, and 5%, respectively [18].

Other reported associated vascular anomalies include a cervical aortic arch, right-sided aortic arch, cervical origin of the SA, persistent trigeminal artery, persistent proatlantal intersegmental artery, bovine origin of the left ICA, aberrant right SA, and absence of the ipsilateral ECA or ICA [2, 3, 6, 8, 11–13, 16]. Table 1 presents previous reports of variations associated with an absent CCA [2, 8, 11–13, 16]. To our knowledge, this is the first report of an accessory MCA in a patient with an absent CCA. Although an accessory MCA is a relatively common vascular variation with a reported prevalence of 0.03–2.7% [15], the existence of a left accessory MCA in the present case may have been incidental because it was located on the contralateral side.

**Table 1:** Previously reported cases of absent CCA associated with arterial variation (2001–2025)

Author (year)	Age/Sex	Side	Associated arterial variation
Rossitti S and Raininko R (2001) [13]	40/F	Left	persistent trigeminal artery
Purkayastha S, et al. (2006) [12]	1/F	Left	right subclavian artery with a cervical origin
Cao YH, et al. (2011) [2]	42/M	Left	persistent proatlantal intersegmental artery
Guha S, et al. (2016) [8]	8/M	Right	cervical aortic arch
Uchino A, et al. (2018) [16]	45/M	Right	aberrant right subclavian artery
Pérez-García C, et al. (2018) [11]	64/F	Left	bovine origin of the left internal carotid artery
Present case	63/F	Right	left accessory middle cerebral artery

In a review of 87 cases involving CCA absence, 13.69% were associated with cerebral aneurysms [17]. Several reports describe endovascular treatment of aneurysms in patients without a CCA [9]. During such endovascular treatment, the CCA may be used as an access route to the aneurysm; therefore, it is important to be aware of this rare variation before the procedure.

## Conclusions

The findings from this case indicate that although CCA defects are asymptomatic, anatomical knowledge and preprocedural imaging are important because they can have a significant impact on planning and outcomes of neurosurgical and endovascular procedures.

## Data Availability

No datasets were generated or analysed during the current study.
